# Evaluation of the new rural cooperative medical system in China: is it working or not?

**DOI:** 10.1186/1475-9276-7-17

**Published:** 2008-07-01

**Authors:** Hassan H Dib, Xilong Pan, Hong Zhang

**Affiliations:** 1 Department of Health Policy and Management, School of Public Health, Peking University Health Science Center, 38 Xue Yuan Road, Beijing, PR China

## Abstract

**Background:**

To prove the possibility of implementing the New Rural Cooperative Medical System (NRCMS) at different levels with a premium funding according to their economic level in developed and less developed areas in Guangdong province, and study the insurable inpatients in different types of regions, taking into account limitations of indemnities and loss ratios.

**Method:**

All data samples were randomly collected from the NRCMS Department, Guangdong Province. Gross domestic product (GDP) at 10000 Yuan per capita was employed to divide Guangdong into two economic levels: (1) economically developed & (2) less economically developed regions. A descriptive analysis about tendency of raising premium and reimbursement ratios of common fund was performed with independent samples and t-test as well as implementing a model to evaluate the differences in premium contribution differences in co-payments, thresholds, and rebates. Also, a qualitative study measured several economic factors to evaluate farmers' financial and social potency in contributing to the NRCMS.

**Result:**

A higher GDP per capita were found within economically developed regions (p < 0.05) than in less developed areas, with higher tendency for funding capacity and average funding capability in villages and towns within economically developed regions (p < 0.05) than in economically less developed. Maximum benefits between two regions in medical insurance coverage showed significant difference (p < 0.05); differences between basic medical insurance coverage between two regions was insignificant (p > 0.05); nevertheless, economically developed regions showed higher threshold and rebates with less co-payments in the economically developed than less developed.

**Conclusion:**

Despite some loop holes in the NRCMS, the system is workable, but needs more strengthening by encouraging farmers' participation into NRCMS with a necessity to implement a new reimbursement payment system by health care providers. In addition it is proposed that for maximum benefits another premium funding should be secured.

## Background

With the introduction of New Rural Cooperative Medical System (NRCMS) in 2003, a significant improvement in China's rural health sector was seen [1]. However, this sector is still facing up with many difficulties, such as shortages in professional staff (GPs, specialized doctors and nurses), technological equipments and appropriate facilities, mostly lying in the mid-west, south-west and north-west regions [2-4]. Many regions are disadvantaged by lack in the fundamental rural health care i.e., low levels of medical insurance coverage and poor reimbursement mechanisms causing the suffrage of farmers from high medical costs with poor access to medical care [5].

For NRCMS-enrolled household farmers, clinics and rural hospitals can only provide services and treatments for acute illnesses, and only hospitals above-rural level are able to treat farmers with chronic diseases due to the presence of advanced medical equipments and the availability of wide selection of medicines, and higher reimbursement ratios from hospitalization expenses i.e., insurance co-payments above rural-hospitals is comparatively higher in economically developed than less developed regions; thus, placing heavy expenses on farmers suffering from chronic illnesses for those living in less economically developed regions.

The objective of this article is to determine whether the current funding model for the NRCMS-supported by the provincial, city, county level governments, and partial contribution from farmers- is considered appropriate and attractive, especially for low income regions in rural China.

## Historical overview

Post 1949, the so called 'socialist medical cooperation centers' (SMCCs) were erected in the urban and rural areas and operated by the 'bare-foot doctors'. However, with the diminish in state funding and inability of bare-foot doctors to cope with the increase in demand for a better health care quality for the rural population, these factors contributed to the collapse and disappearance of the SMCCs or became individually owned. Between the gradual disappearance of the SMCCs and the bare-foot doctors, and the full operation of the CHC system (1978–1998) in the urban areas, the rural areas suffered from severe shortage of health facilities and medical professionals responsible for providing basic medical services for a huge rural population [5].

Notwithstanding the collapse of the old rural economic system and the emergence of the sublet system by the beginning of 1980, the old Rural Cooperative Medical System (RCMS) was developed. Under this system, the funding of the rural health care depended on a pre-payment plan, supported by three routes: (i) premiums between 0.5 and 2% of yearly income paid per farmer's household to the commune fund system- decided by the plan's benefit composition and the local community's economic level; (ii) every village donated a specific proportion of income from shared agricultural production or rural ventures to a welfare fund, according to state guiding principles; and (iii) government funding was utilized to defray health workers and acquire medical equipment [6,7].

By the 1980s, China started moving from a centrally planned to a market economy i.e., transforming from collectivity system to what became known as a 'household system', which destabilized the financial foundation of the cooperative medical system and led to the downfall of old RCMS schemes in many rural areas; by 1993 the medical insurance coverage in the rural areas dropped to 12.8% [7]. Meanwhile, the central government took further measures and ceased their financial support towards hospitals and other health institutions, causing disruptions in hospitals' revenues. This forced hospitals to search for several routes to counterbalance the financial returns such as the adoption of the bonus system and regulated prices policies. This alteration in the health care providers' behaviour forced physicians, indirectly, to provide unnecessary care. As a result, the percentage of profits earned by physicians increased per month, drove the percentage of medical fees to increase as well as causing a huge burden on the majority of uninsured population in China (79% in rural and 44.8% in urban, according to 2003 statistics; 44.8% are paid out-of-pocket in the urban areas, and 87.3% in the rural areas, according to 1998 survey [8-11].

## Method

All data were collected from the department of NRCMS in Guangdong Province, government economic division. Data was extracted randomly by taking 10000 RMB GDP as basic level, where farmers earning above 10000 RMB per capita are designated as those living in the economically developed regions, and others earning below 10000 RMB are designated living in the economically underdeveloped regions, Table 1[12].

**Table 1:** GDP per capita in various rural regions in Guangdong (Yuan)

Economically developed regions	GDP per capita	Economically less developed regions	GDP per capita
Dongguan	71995	Heyuan	5186
Guangzhou	56271	Meizhou	7012
Foshan	47658	Qingyuan	7484
Zhongshan	44006	Shanwei	8483
Zhuhai	41048	Jieyang	9067
Jiangmen	21647	Zhanjiang	9733
Mean ± SD	47104.1429 ± 15265.6165	Mean ± SD	7827 ± 1492.0638

*Exchange rate during the last 2 years: 1 USD = 8 RMB Jan 2006; 1 USD = 7.46 Sept 2007

**Figure 1:**
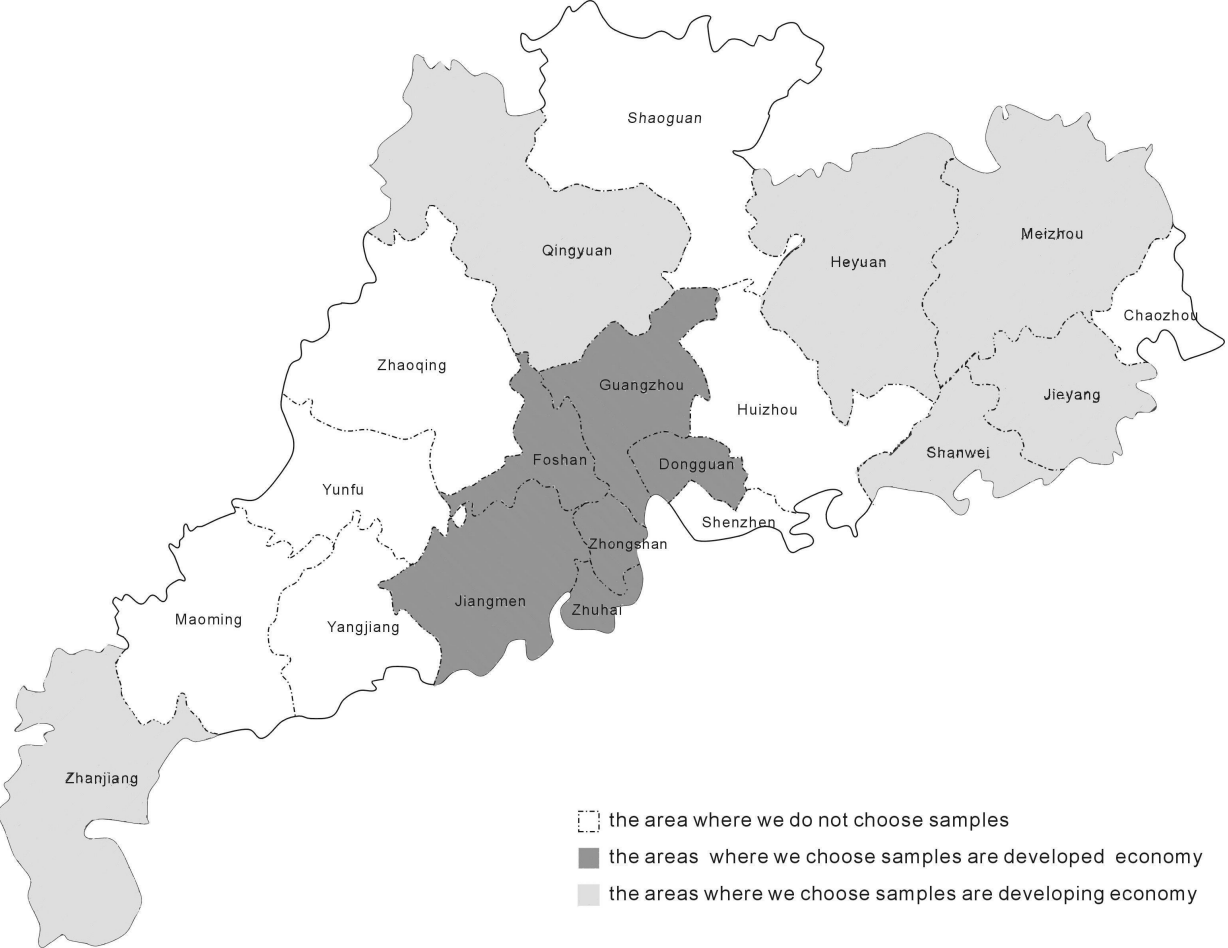
showing both developed and less developed areas in Guangdong province where samples were extracted and were compared.

In Guangdong province there are 21 cities and among them 11 are developed and 10 cities are under-developed. Six cities were chosen randomly for our sample from the developed and under-developed ones. The economically developed regions included: Dongguan, Guangzhou*, Foshan, Zhongshan*, Zhuhai* and Jiangmen, and economically underdeveloped regions included: Heyuan, Meizhou, Qianyuan, Shanwei, Jieyang, and Zhanjiang* (Figure 1).

A stratified random sampling based on different economic factors illustrated in Additional file 1. The software, SPSS v13.0 was used to perform the descriptive analysis, including independent-samples *t*-tests. Cronbachs' alpha was used showing α = 0.84, which has a good reliability for our study. Furthermore, the method applied to analyze the causes in this study showed that the questionnaire's form was reliable and efficient. Kaisex-Meyer-Olkin (KMO) was utilized to test whether the quantity of participators were enough or not, and Barlett method was used to test the suitability of the model for the causes in our study.

### The Estimation Model

We constructed dummies to evaluate the capability of a farmer to contribute to the NRCMS and observe its viability. We took the average cost of consulting a general physician as a dummy variable that equals one if the consultation is at 5 RMB and zero otherwise; consulting a medical specialist is a dummy variable equal to one if the rate is at 10 RMB and zero otherwise, a dummy variable for-average purchase of drugs for a common cold-is equal to one if the rate is at 100 RMB and zero otherwise; a dummy variable is equal to one if the purchase of drugs for hypertension (as a chronic disease) 250 RMB and zero otherwise; a dummy variable for basic tests required before hospitalization- as required by the China Ministry of Health- is equal to one if the cost is at 700 RMB and zero otherwise; a dummy variable for average cost for hospitalization for a chronic illness is equal to one if the cost is at 1000 RMB and zero otherwise; and a dummy variable for the "additional expected utilization of health services" is equal to one if is at 500 RMB and otherwise zero.

The average costs of medical treatment in the three types of health institutions those listed in Table 2 &3 are taken from pharmacies, financial departments of these institutions. Consultation fees and medication coverage and reimbursement ratios are dependent on the type of premium, forcing patients to finance the purchase of their own medications through co-payments.

**Table 2:** Reimbursement ratios of common fund at different hospitals' level between economically developed and less developed regions

Regions	Reimbursement ratios for hospitalization expenses (Mean ± SD)		
	
	Clinics	County level hospitals	Hospitals over county level
Economically less developed regions	45.50 ± 4.97	38.50 ± 4.11	30.00 ± 2.36
Economically developed regions	54.92 ± 11.87	44.00 ± 11.23	34.54 ± 13.89
P-value	0.46	0.11	0.005

Reimbursement values are represented in RMB.

* Exchange rate during the last 2 years: 1 USD = 8 RMB Jan 2006; 1 USD = 7.46 RMB Sept 2007

**Table 3:** premium and threshold benefits coverage for hospitalization expenditures at each experimental county

Regions	Minimum premium medical insurance coverage (Mean ± SD)			Threshold benefit insured coverage
		
	Clinics	County hospitals	Hospitals above county level	
Economically less developed regions	188.00 ± 55.73	320.00 ± 63.25	480.00 ± 122.93	5930.00 ± 2061.31
Economically developed regions	296.15 ± 116.30	503.85 ± 165.15	742.31 ± 165.15	22846.15 ± 20099.11
P-Value	0.118	0.054	0.132	0.005

Insurance costs are represented in RMB.

* Exchange rate during the last 2 years: 1 USD = 8 RMB Jan 2006; 1 USD = 7.46 RMB Sept 2007,

(1) ω ∝ [(σ + Φ) ∝ π]

Where σ = Farmer's raising premium; Φ = government premium;

ω = Threshold benefit insured patient coverage; π = insurance premium

(2) ∝ = Ω × κ

Where ∝ = co-payments; Ω = treatment costs; κ = reimbursement ratio

From equation (1), σ is increased by increase in farmers' household contribution to the NRCMS; in parallel with further adjustment of Φ according to yearly inflation rate, their capitation is proportional to the increase in the premium π; as a result the threshold benefit insurance coverage is increased simultaneously as seen in Table 3. In the less economically developed areas, farmers are contributing with a low premium to the NRCMS (Table 4), providing farmers with 50% reimbursement returns as shown in (Table 2) in comparison to developed region. From equation (2), ∝ is the gap where farmers need to finance their hospitalization in the form of co-payments. The co-payments are affected by the minimum premium level of farmers from less developed region they have invested in the NRCMS. As seen from Table 1, there is a 6 times difference in the GDP per capita between the two regions in Guangdong province, which is affecting farmers' abilities to contribute to NRCMS. The difference could also be detected in both the survey data collected from both regions as seen in (see Additional file 1).

**Table 4:** Ability of raising premium from household farmers who participated in the NRCMS per year (RMB)

Contribution to funds	Economically less developed regions (Mean ± SD)	Economically developed regions (Mean ± SD)	P-Value
Household farmers	12.50 ± 3.54	58.77 ± 42.57	0.002
Villages and towns	3.00 ± 1.41	24.77 ± 13.43	0.005
Counties	6.20 ± 2.30	29.58 ± 22.62	0.020
Cities	3.80 ± 1.39	12.00 ± 11.18	0.000
Average funding per household	48.50 ± 4.12	117.31 ± 70.43	0.002

* Exchange rate during the last 2 years: 1 USD = 8 RMB Jan 2006; 1 USD = 7.46 Sept 2007

As for Ω, symbolizes hospitalization cost that varies and is dependent on the type of the disease whether it is in the acute or chronic state. ê signifies the reimbursement ratio, which is dependent on the type of premium insurance coverage; i.e., the higher is the contribution to the premium from farmer's side, the better the reimbursement ratio and less are the co-payments paid by the farmer (see Additional file 2). Hence, taking the GDP per capita for each city in the less developed region, and the 10,000 RMB used as a standard GDP per capita for the two regions, there is a need for farmers to increase their contribution to the premium from 10 RMB to 15 RMB, and at the same time Guangdong government sustaining its contribution at 25 RMB or increasing it according to yearly inflation rate, then the rebates levels will increase at least to 75%, and the threshold benefits of insurance coverage will be increased; thus, providing enough funds for the system to work. As for the economic developed region, farmers in that region have relatively higher threshold benefits insurance coverage and higher rebates than the less developed regions due to their relatively higher contribution to NRCMS. Comparing their GDP per capita, and the 10,000 RMB GDP per capita taken as a standard, farmers in that region are able to contribute more to the NRCMS; thus, providing further support to the funds to keep the system working, and that is achievable by increasing farmers' premium up to 20 RMB and at the same time the Guangdong's government increasing its contribution to 20 RMB, which will lead to more support for the funds with higher threshold benefit insurance coverage, with less co-payments from farmers need to pay and more rebates without affecting the performance of the system, but rather maintaining its workability.

## Results

The Guangdong government is paying 10 RMB for developed regions and 25 RMB for underdeveloped region to obtain the premiums coverage treatment at the community health centers (CHCs), county hospitals and above county hospitals as shown in Table 3. If we take each region-(developed regions > 10,000GDP per capita < less developed regions)-as illustrated in Table 1, there is statistical significant difference only in the premiums benefits coverage for hospitalization expenditures observed above-county hospitals between the developed and less developed regions due to their ability to deliver advanced treatments (P < 0.005) Table 3. Also, the significant differences lies in the threshold coverage between the two regions (P < 0.05). This wide difference in the threshold is dependent on the level of contribution of households to the NRCMS in both economic regions (Table 3); hence, low threshold reflects higher co-payments due to low reimbursements ratios at various health institutions within the experimental county sites (see Additional file 2). So, when we measure the co-payments rested upon farmers, it still reveals a huge financial burden inflicted on the farmer household in the less developed regions in comparison to their GDP per capita.

From equation 2 applied for the population residing in the less developed region, as the premium increases rebates decreases and co-payments increases as seen from Table 2, 3 & (see Additional file 2); resulting in higher farmers' co-payments for hospitalization in the less developed region. This is attributed to low contribution of farmers and higher contribution from the governments to the NRCMS P < 0.002 (Table 4); thus, delivering lower rebates from the funds (according to equation 1 and 2). Also, applying equation 2 for the developed economic region, as premiums increases the rebates increases and co-payments decreases relatively more than it is existing in the less developed regions (Table 2, 3 & see Additional file 2); this is attributed to the higher contribution of farmers to the NRCMS P < 0.002 (Table 4) and less input from the government; thus, delivering higher rebates from the funds (according to equation 1 and 2).

Raising premiums from household farmers, villages and towns, counties, and cities the average funding per household in the two economic regions shows significant differences P < 0.05 to 0.001 (Table 4); this is being observed as well from the social and economic analysis observed (see Additional file 1) due to wide disparity in the GDP per capita in the two economic regions.

If we take each city's GDP in the less developed and developed economic regions as shown in Table-1-taking 10,000 RMB as standard- shows there is wide deficit in the GDP per capita in the less developed economic region compared to the developed economic region causing difficulty to raise relatively higher premiums from the less developed regions, more than what they are paying at the present, in order to contribute more to the funds.

## Discussion

As seen from our data analysis, the disparity in GDP per capita between the two economic developed and less developed regions has influential effects on farmers' enrolment into the NRCMS. It is obvious that farmers living in the less developed region have limited capability of paying more to the premium. In fact, there is a disconnection between providing sufficient incentives, due to the very low returns, when a person require to utilize the insurance coverage, and at the same time providing sustainability for the funds in order for the system is able to work. Three factors have influence on this dis-connectivity 1) lack in the basic knowledge, especially among aged ones, about the cost benefit of medical insurance coverage and their understanding of the social insurance industry in maintaining their own continuous participation. 2) Absence of strong promotion targeting NRCMS cost-benefits, advantages, compensation methods, and provisions. In China, farmers believe that paying certain fees for medical treatment is only necessary when they are in need to see a doctor, which is the opposite case when they are in good health, and consider it as a waste to get enrolled in the NRCMS-a cultural point of view- necessitating for a wide publicity about enrolling into NRCMS and its benefits.

3) There is lack of sufficient number of high qualified doctors and other health professionals, as well as absence of high revenues in the rural areas, making it difficult for health institutions to purchase advanced medical equipments; thus, driving away the rural population to search for medical treatments in the above-county level hospitals bringing furthermore higher health service costs, higher co-payments, and less rebates. Also, the variation in the medical reimbursements between developed and the less developed areas have an effect on farmers' access to medical services. For example, when farmers feel their hospitalization days will be prolonged and the treatment costs will be high, there is a demand for an early discharge based on farmer's request, which is not un-common phenomenon in order to avoid further higher payments especially for those suffering from chronic diseases. The average hospitalization expenses for patients with chronic diseases is approximately 12,000 RMB per inpatient episode [13], but in the less economically developed regions the threshold benefits for hospitalization expenses reaches 6000 RMB per inpatient episode. With threshold benefits set at this low level, despite the existence of a large funding reserve (20 Billion RMB per year) equalizing 12 months of premiums, the NRCMS-enrolled farmers do not receive reimbursements commensurate according to their payments [14]. These policies undermine farmers' confidence in the NRCMS, causing their hesitancy to participate and/or withdrawal from this insurance coverage.

Researchers [15] have acknowledged that income is an important factor influencing farmers' decision to join the NRCMS, even though the premium represented a very small portion of household income. Both income and health status are factors affected consumers' use of health services [15]. The richer, sicker enrollees receive greater net benefits (NB)-NB is the discrepancy between the value of services an individual receives and the summation of premium and co-payment an individual pays out-of-pocket (NB is totally different from the 'net benefit' utilized in cost-benefit analysis and cost-effectiveness analysis), showing the poorer healthier participants financing and supporting indirectly the rich sick. Finally, well-off farmers receive greater benefits from CBI due to their relatively low premiums and high co-payments at every level of the health system [15].

## Summary of latest update on new rural CMS

During 2005 Guangdong province has succeeded in establishing the NRCMS in approximately 120 counties. This NRCMS covered almost every village; 50.5% of Guangdong residents received approximately 1.08 billion Yuan in reimbursements [16]. Also, 77 counties (66%) used the new cooperative financial compensation system centered on care for chronic diseases. Guangdong's government incorporated residents, living in less economically developed regions, into the cooperative medical system for those earned an annual income lower than 1500 RMB within the period 2002 to 2006 [17] by financing the NRCMS with 0.6 billion RMB for reimbursements [17]. By the end of 2006 most towns and villages in Guangdong Province have finalized in setting up their NRCMS, and more than half of its population were covered by this system [18], and more than 30 million people have participated in the NRCMS, and have extended its coverage to 61% of Guangdong residents [19]. The Guangdong Government also has provided additional 10 billion Yuan for the establishment of NRCMS relief fund.

At the national level, in 2003 approximately 304 counties were involved in the experimental project, and by 2004 the number had increased to 333 counties. By 2005, there was at least one experimental county in each region [20]. In September 2005, approximately 671 counties nationally implemented the NRCMS experimental project, involved approximately 177 million out of approximately 745.44 million farmers [21,22]. On 10^th ^January 2006, the Ministry of Health with six other ministries announced an acceleration of the NRCMS experimental project [23], and by September 2006, approximately 1433 counties were enrolled in the project, which represented 50.1% of counties nationwide [23].

The Chinese government projection for the 2008 is to expand the NRCMS project and be able to cover the whole rural population by 2010. In Shaanxi Province, for example, the NRCMS has covered more than 12 million farmers since 2003, or 44.5% of the rural population, with a total fund of 612 million Yuan (approximately US$78.6 million) [24]. By the beginning of 2007, approximately 406 million farmers or 45.8% of the total rural population in China has joined the NRCMS [24].

The new cooperative medical system is an essential insurance type for the rural population, but requires further strengthening and rectification on several aspects to maintain its performance. It is important to implement a new health policy able to develop the health service industry in the rural areas [25], same problem facing many countries such as in Indonesia, India, Thailand, African countries, etc. There is a necessity for the provincial and local governments to increase their promotion about the advantages of joining the NRCMS through various methods, increase insurance benefits and establish an appropriate level of compensation; enforce on the newly specialized physicians to work for a period of time in the rural areas, improve doctors' standard and train those who are willing to work in the rural areas. By these strategies the rural population will have the ability to 'see a doctor' without the need to seek medical services at the above-county level hospitals, at affordable fees. Furthermore, there is a necessity for the governments (provincial and local) to increase their premium contribution into the NRCMS in order to maintain their support for the rural population in the less developed economic region. By doing so, there will be persistent sustainability of the funds leading to continuity of the system. However, the long run objective and the most practical solution lies in improving the economic development of the rural areas, which will increase the GDP per capita of the less developed regions and provide the rural population with more capability to contribute to the NRCMS premium.

## Conclusion

There is disparity in the contribution methods to the premium between the developed and underdeveloped economics regions in Guangdong province, as well in the ceiling or the threshold coverage, and in the hospitalization rebates. Nevertheless, despite some loop holes in the system, the system is workable and more of the rural populations are enrolling into this insurance coverage. As for the maximum benefits other premium funding should be secured.

## Competing interests

The authors declare that they have no competing interests.

## Authors' contributions

HHD designed the study, analyzed and interpreted data, and drafted the manuscript and finalized writing it, XLP supervised the study, and provided statistical advice throughout the study, HZ involved in performing the surveys, collected data from the Guangdong team and provided detailed commentary. All authors read and approved the final version of the manuscript.

## Supplementary Material

Additional file 1 Statistical economic evaluation/household survey. The data provided represent the economic and social statistical analysis for the developed and less developed regions in Guangdong province Click here for file

Additional file 2 payment fees. Dummies showing payment fees of hospitalization and difference of farmers' co-payments required to pay for hospitalization expenditures at each experimental county Click here for file

## References

[B1] Lei Yu Lan (2007). Guangdong province health affairs meetings report. http://www.gdwst.gov.cn/html/wsjb/200703202115.html.

[B2] Hassan DibH, Shen Yan Hong, Zhao Bin, Shen Hongliang (2007). Health Care cost in China: Need for intervention. Journal of Health Management.

[B3] Du XW, Du HY (1999). Mechanism for adjusting health services prices. Chinese Health Economics.

[B4] Li SX (1997). The problems of hospital reimbursement and related suggestions. MIMEO Jinan: Institute of Social Medicine and Health Policy.

[B5] Pan Xilong, Hassan DibH, Wang Xiaohang, Zhang Hong (2006). Analysis of Service Utilization in Community Health Centers Compared with the Local Hospitals in China: a comparative analysis. BMC Health Services Research Journal.

[B6] Liu YL (2004). Development of the rural health insurance system in China. Health Policy and Planning.

[B7] Chen CC (1989). Medicine in rural China: a personal account.

[B8] Ministry of Health (PRC) (1994). Research on national health services- an analysis report of the National Health Services Survey in 1993.

[B9] Ministry of Health (PRC) Reports on the 1998 National Health Services Survey results.

[B10] China Ministry of Health (PRC) (2003). Reports on the 2002 National Health Services Survey results.

[B11] Gao Jun, Qian Juncheng, Tang Shenglan, Erikblas Boeriksson (2002). Health equity in transition from planned to market economy in China. Health Policy and Planning.

[B12] Guangdong provincial government (2006). Guangdong economic development outline. Online.

[B13] Jiang R (2003). An investigation on rural health security after the agricultural tax reform. Chinese Health Economics.

[B14] (2005). National economic and social development statistics in Guangdong. http://www.gdstats.gov.cn.

[B15] Wang H, Yip W, Zhang L, Wang L, Hsiao W (2005). Community-based health insurance in poor rural China: the distribution of net benefits. Health Policy and Planning.

[B16] Mu Xuequan Survey: China's new medicare system relieves burden of rural residents. http://www.chinaview.cn.

[B17] Guangdong provincial government (2005). Data of sample survey of 1% of the national population (No. 2) in Guangdong. http://www.gdstats.gov.cn.

[B18] Guangdong provincial government (2005). Data of sample survey of 1% of the national population (No.1) in Guangdong. Online.

[B19] Qingyue Meng Developing and implementing equity-promoting health care policies in China. A case study commissioned by the Health Systems Knowledge Network, 2007.

[B20] Lai Ruinan, Yu Rongrong, Zhou Yudong (2006). Application of payment method (paying by per person) for new type of rural cooperative medical service. Chinese Health Services Management.

[B21] (2005). National new type of rural cooperative medical experiments achieved significant results.

[B22] (2005). National economic and social development statistics of People Republic of China. http://www.stats.gov.cn.

[B23] Ministry of Health and other six ministries 'the notice to accelerate the work of experimentation on new-type of cooperative medical service in rural areas'. http://wwww.chinavalue.net/wiki/showcontent.aspx?titleid=71210.

[B24] Feng Tao (2007). China rebuilding rural cooperative medicare system. http://www.chinaview.cn.

[B25] Liu Y, Rao K (2006). Providing health insurance in rural China: from research to policy. J Health Polit Policy Law.

