# Supplementary material for: Evaluation of the new rural cooperative medical system in China: is it working or not?
Source: Int J Equity Health. 2008 Jul 1;7:17. doi: 10.1186/1475-9276-7-17 (PMC2459170; doi:10.1186/1475-9276-7-17)
Supplement: Additional file 1 — Statistical economic evaluation/household survey. The data provided represent the economic and social statistical analysis for the developed and less developed regions in Guangdong province [file 1475-9276-7-17-S1.doc]

Additional File 1

Table –A1 Statistical economic evaluation⁄ household from both developed and underdeveloped economic regions

| **Source of income/household** | **Developed regions** | **Underdeveloped regions** |
| --- | --- | --- |
| -Agricultural machine ownership | 79% | 57% |
| -small factories Ownership | 16% | 12% |
| -Farming and Herding | 24% | 63% |
| -Fishery | 7% | 3% |
| -Labor | 20.5% | 15.5% |
| -Transportation ownership | 13.5% | 6% |
| -Small shop ownership | 8% | 2% |
| -Small restaurant ownership | 11% | 1.5% |
| **Number of people living**  **per household** | 5 | 7 |
| **Number of people with Educational level ⁄household** |  |  |
| -No education | 52% | 78% |
| -Primary school | 21% | 11% |
| -Junior school | 15% | 8% |
| -High school | 8% | 2% |
| -University level | 4% | 1% |
| **Total income⁄ household/** year | Mean 14326 RMB* | Mean 8334 RMB* |
| **Reasons for number of migrated people⁄ household from villages to cities** |  |  |
| -Seeking university education | 21% | 15% |
| -Seeking better income | 73% | 83% |
| -Daily migration | 6% | 2% |

* Exchange rate during the last 2 years: 1 USD = 8 RMB Jan 2006; 1 USD= 7.46 Sept 2007
