# Supplementary material for: Evaluation of the new rural cooperative medical system in China: is it working or not?
Source: Int J Equity Health. 2008 Jul 1;7:17. doi: 10.1186/1475-9276-7-17 (PMC2459170; doi:10.1186/1475-9276-7-17)
Supplement: Additional file 2 — payment fees. Dummies showing payment fees of hospitalization and difference of farmers' co-payments required to pay for hospitalization expenditures at each experimental county [file 1475-9276-7-17-S2.doc]

Additional File-2

Table-A2 Dummies showing payment fees of hospitalization and difference of farmers’ co-payments required to pay for hospitalization expenditures at each experimental county

| Regions | Dummy hospitalization fees | | |
| --- | --- | --- | --- |
|  | Clinics | County hospitals | Hospitals above county level |
| Average Consultation fees | 4RMB | 6RMB | 10RMB |
| Average Treatment cost | 100RMB | 600RMB | 1500RMB |
| Average Drugs Fees | 100RMB | 250RMB | 1000RMB |
| Average Test fees | 45RMB | 500RMB | 700RMB |
| **Economically less developed regions** |  |  |  |
| Reimbursement fees | 682.50RMB | 577.50RMB | 450.00RMB |
| Farmer’s difference payment fees | 682.5RMB | 922.50RMB | 1050RMB |
| **Economically developed regions** |  |  |  |
| Reimbursement fees | 823.80RMB | 660.00RMB | 518.10RMB |
| Farmer’s difference payment fees | 676.2 RMB | 840.00RMB | 981.90RMB |

* Exchange rate during the last 2 years: 1 USD = 8 RMB Jan 2006; 1 USD= 7.46RMB Sept 2007,
